# Patient Mobility in the Digital Era: How Online Service Information from Internet Hospitals Shapes Patients’ Cross-Regional Healthcare Choices

**DOI:** 10.3390/healthcare13050484

**Published:** 2025-02-23

**Authors:** Yingjie Lu, Luli Shi, Zimeng Wang

**Affiliations:** School of Economics and Management, Beijing University of Chemical Technology, Beijing 100029, China; luyingjie982@163.com (Y.L.);

**Keywords:** patient mobility, online medical service, cross-regional healthcare choices, signaling theory, China online healthcare platforms

## Abstract

**Background/Objectives:** Patients in medically underserved regions often seek cross-regional healthcare for high-quality medical services but face significant barriers due to limited information about providers. Internet hospitals address this gap by offering online consultations, remote diagnoses, and public service information. This study examines how such information shapes patients’ cross-regional healthcare choices. **Methods**: A binary logistic regression model using signaling theory was employed to evaluate the impact of platform-generated signals (e.g., hospital ratings) and patient-generated signals (e.g., review quantity and polarity) on patients’ cross-regional healthcare choices. The experimental data were sourced from a leading Chinese online medical platform, comprising 1901 hospitals and 273,884 patient feedback records. Among these, 216,793 patients (79.16%) sought cross-regional treatment, while 57,091 patients (20.84%) opted for local treatment. **Results:** Platform-generated signals, such as hospital ratings (B = 0.406, *p* < 0.01) and patient-generated signals, including review quantity (B = 0.089, *p* < 0.01) and polarity (B = 0.634, *p* < 0.01), significantly and positively influence patients’ cross-regional healthcare choices. Disease severity and local medical resource availability moderated these effects: Patients with severe conditions rely less on hospital ratings (B = −0.365, *p* < 0.01), while those in resource-limited areas depend more on hospital ratings (B = −0.138, *p* < 0.01) and review quantity (B = −0.029, *p* < 0.01) but less on review polarity (B = 0.273, *p* < 0.01). **Conclusions:** These findings offer actionable insights for policymakers and platform developers to optimize online healthcare services, facilitating informed cross-regional healthcare decisions and advancing healthcare equity in the digital era.

## 1. Introduction

The rapid rise of internet healthcare services has significantly expanded access to high-quality medical resources, especially for patients in medically underserved regions [1]. For individuals unable or unwilling to visit healthcare facilities in person, internet healthcare offers efficient services such as online consultations and remote diagnostics, effectively reducing the individual’s time and financial burdens [2,3,4]. Moreover, the importance of internet healthcare cannot be overlooked for patients planning to seek in-person treatment. With the widespread adoption of internet technology, the accessibility of medical service information and doctor–patient communication has been significantly enhanced. Recent studies have demonstrated that Internet healthcare has not only transformed traditional face-to-face communication models but also reduced information asymmetry by providing patients with more comprehensive access to medical service information [5]. This shift has empowered patients with greater autonomy and decision-making capabilities, thereby promoting a more patient-centered approach to healthcare communication and engagement.

However, in traditional healthcare settings, patients often face significant challenges in assessing the capabilities and quality of services offered by hospitals, which limits their ability to make informed decisions. This issue is especially challenging for patients seeking care in distant locations. The lack of information about out-of-area hospitals creates uncertainty about whether traveling for treatment is worthwhile, complicating their decision-making. The emergence of Internet healthcare services has the potential to address this challenge by providing patients with a critical source of information. Through access to publicly available online service information, patients can evaluate the performance, reputation, and quality of healthcare providers, enabling them to make more informed health-related decisions.

However, while the existing research on online medical services has primarily focused on online doctor–patient interactions, such as behaviors, characteristics, motivations, and benefits, there remains a significant gap in understanding how publicly available online service information influences patients’ cross-regional healthcare choices. Specifically, little is known about which types of online information are most impactful in reducing information asymmetry and guiding patients’ decisions to seek care outside their local regions. This study aims to fill this gap by exploring the role of publicly available online service information, provided by internet hospitals via online medical platforms, in reducing information asymmetry for cross-regional medical decisions. Specifically, it seeks to identify which types of information have the most significant impact on these decisions. Signaling theory, which addresses the challenges of information asymmetry, offers a robust theoretical framework for this investigation. Information asymmetry arises when one party (e.g., healthcare providers) possesses more information than another party (e.g., patients). To bridge this gap, signaling theory posits the informed party can convey credible signals to reduce uncertainty and enhance trust. Online service information from Internet hospitals acts as these signals, enabling patients to assess the quality and reliability of healthcare providers. By applying signaling theory, this study examines how these signals alleviate the consequences of information asymmetry and facilitate informed decision-making in cross-regional healthcare contexts. Consequently, the core research questions of this study are as follows:

*How does the online service information provided by internet healthcare platforms influence patients’ cross-regional healthcare choices, and more precisely, which types of online signals play a critical role in shaping these decisions?*

By developing an analytical framework grounded in signaling theory, this research identifies key types of signals and evaluates their influence on patients’ cross-regional healthcare choices. The insights from this study will enhance our understanding of how internet healthcare platforms influence patient mobility. They will also contribute to improving policy development and platform design in the digital healthcare era.

## 2. Literature Review and Hypothesis Development

### 2.1. Patient Cross-Regional Mobility

The cross-regional mobility of patients for medical treatment is a widespread global phenomenon that is particularly prevalent in regions such as the European Union (EU), the United Kingdom, and China [2,3,4,6,7,8]. To provide a broader perspective, similar mobility patterns and challenges have been reported in the Gulf Cooperation Council (GCC) countries, where disparities in healthcare quality and accessibility play a crucial role in shaping patient decisions [9]. The existing studies widely recognize the uneven distribution of medical resources as a core factor driving this phenomenon [4,10]. A survey in the EU reported that 71% of patients pursued cross-border care due to unavailable treatments in their home country, whereas 53% sought higher-quality services [6]. These findings suggest that the lack of local medical resources and the desire for higher-quality medical services are the primary drivers of cross-regional mobility.

The cross-regional healthcare-seeking behavior of patients is influenced by a combination of individual characteristics and structural factors based on Andersen’s behavioral model of health services [4,8]. Individual factors include demographics (e.g., age, gender, education), socioeconomic status (e.g., income, health beliefs), and disease-related attributes (e.g., severity) [2,4,11,12,13,14,15]. Structural factors refer primarily to factors within the regional medical system, including medical resource availability, service quality, and medical insurance coverage [12]. The recent research has further expanded Andersen’s model by categorizing factors that influence cross-regional medical choices into “availability” (e.g., the sufficiency of hospitals, available beds, and medical professionals), “affordability” (e.g., insurance coverage and reimbursement policies, income levels), “accessibility” (e.g., geographical distance, transportation), and “economic level” (e.g., financial ability to afford travel expenses) [12,13]. Additionally, patients may also consider their familiarity with the target region and their perception of medical quality when selecting cross-regional treatment [13,14,16]. Overall, cross-regional healthcare choices are influenced by multifaceted interactions of individual, systemic, and policy-related factors [2,11,17].

Despite these studies exploring the multidimensional and complex influencing factors of cross-regional patient mobility, research indicates that patients often face challenges in making rational decisions due to factors such as limited access to information, the unreliability or unavailability of data, and individual capability constraints [12,18]. First, patients seeking cross-regional medical care face significant challenges due to restricted access to essential information [18]. Without the support of familiar local networks and guidance systems, they struggle to obtain comprehensive and accurate details about medical institutions in other regions, including service quality, processes, and treatment outcomes. The absence of clear information decreases their ability to make informed choices, frequently resulting in the selection of suboptimal healthcare options [19]. Second, patients are often confronted with flawed information that is overloaded, untrustworthy, and poorly presented, making it difficult to assess the quality of healthcare providers effectively [12]. Despite evidence that comparative data on various aspects of available hospitals can help patients better choose medical providers, this information is rarely applied in real-life situations. Patients may perceive this information as irrelevant or too complex to understand, be unwilling to dedicate further time to comparing options, or lack the capacity to judge complex information. Finally, individual capability constraints are critical factors that limit patients’ ability to make rational decisions. Rational healthcare choices require patients to possess high levels of health literacy (including the ability to access, process, and understand health information) and numeracy skills (the ability to apply numerical information in health management) [12]. However, many patients lack these essential competencies, which makes the complex decision-making process difficult for them when selecting medical institutions.

Therefore, addressing the challenges of limited information accessibility, clarity, and trustworthiness is essential for empowering patients to make more rational and informed decisions. To achieve this, we must focus on improving the availability of reliable data and presenting it in user-friendly formats. By doing so, we not only empower individuals to make better informed decisions but also facilitate the optimization of patient mobility and ensure equitable access to high-quality healthcare across regions.

### 2.2. Signaling Theory for Online Service Information from Internet Hospitals

Signaling theory is a widely adopted analytical framework for understanding how consumers, confronted with information asymmetry, evaluate the quality of products prior to purchase. Consumers typically rely on various signals, such as product introductions or word-of-mouth recommendations, to infer product quality, establish trust, reduce perceived risk, and ultimately influence their purchasing decisions. Especially for goods characterized by high levels of information asymmetry, such as experience goods and credence goods, consumers often cannot evaluate quality solely on the basis of observable characteristics and thus must rely heavily on external signals to make informed judgments about product quality.

When patients consider seeking medical treatment in distant hospitals, they often cannot visit the hospital in person. Instead, they gather information about these hospitals from various sources to evaluate their medical services. On the one hand, patients can obtain internal signals provided by hospitals from their official websites, promotional materials, and news reports. However, such information is typically concise and homogeneous and lacks comparative data based on uniform standards, which poses significant challenges for patients in making comparative decisions among multiple hospitals. On the other hand, patients may turn to external signals, such as reviews shared by family and friends or word-of-mouth recommendations from local social media. Unfortunately, given that distant hospitals are relatively unfamiliar to patients and that friends or family may not have comprehensive knowledge of the target hospital, these external signals are often scarce, making decision-making even more difficult.

In this context, internet hospitals offer a unique solution by providing online medical service information. This serves as a valuable external signal about their services that is both comprehensive and easily accessible and can significantly reduce the cost of searching for information and alleviate information asymmetry when hospitals are chosen for cross-regional medical treatment [20,21]. The external signals provided by internet hospitals can be categorized into platform-generated signals and patient-generated signals [22,23]. Platform-generated signals include comprehensive evaluations, rankings, certifications, and recommendations provided by the online platform itself, which can offer patients an intuitive understanding of a hospital’s overall medical capabilities. Patient-generated signals, on the other hand, consist of feedback and reviews from individuals who have previously utilized the hospital’s medical services. These signals reflect the hospital’s actual performance and reputation from the perspective of its patients, thus providing reliable insights into service quality and patient satisfaction (reflecting the actual patient experience and the hospital’s reputation) [22,24,25,26].

#### 2.2.1. Platform-Generated Signals

Platform-generated signals, derived from the aggregation of diverse hospital-related metrics by online medical platforms, play a critical role in assisting patients with cross-regional medical decisions. These signals combine internal attributes of hospitals—such as grade, size, certifications, specialized departments, medical equipment, and doctor teams—with historical service data available on the platform, including patient interaction records, consultation transcripts, and offline follow-up visits [20,21,24,25]. By consolidating these metrics into comprehensive evaluations, rankings, and recommendations, platforms provide patients with an objective, accessible, and intuitive comparison of hospitals, thereby reducing cognitive overload and facilitating informed decision-making, especially for patients seeking healthcare in unfamiliar regions. Numerous prior studies have highlighted the significance of platform-generated signals in influencing patient behavior. Metrics such as comprehensive recommendations, overall ratings, and rankings have been shown to effectively attract patients, increase online consultation volumes, and enhance doctor performance [27]. These findings suggest that patients rely heavily on such signals when assessing the credibility and quality of hospitals.

When patients consider cross-regional medical visits and turn to online medical platforms for information, platform-generated signals, such as comprehensive ratings and recommendations, play a critical role in shaping patients’ perceptions of hospital credibility [20]. These signals provide an accessible, objective, and reliable basis for evaluating hospitals, reducing the inherent uncertainty and perceived risks associated with cross-regional medical decisions. Furthermore, drawing on health behavior models, it is evident that perceived benefits, barriers, and self-efficacy play a critical role in shaping health-related decisions. Platform-generated signals, such as comprehensive ratings and recommendations, align with these constructs by offering patients clear and actionable information, which strengthens their confidence and reduces uncertainty in cross-regional medical decision-making. Based on the above, it is reasonable to hypothesize that platform-generated signals—such as comprehensive recommendations, aggregated evaluations, and ratings—serve as critical indicators of hospital credibility. These signals significantly influence patients’ cross-regional medical decisions. Thus, we propose the following hypothesis:

**H1:**  *Platform-generated signals, such as comprehensive recommendations, evaluations, and ratings, have a significant positive effect on patients’ decisions about cross-regional medical decisions.*

#### 2.2.2. Patient-Generated Signals

Patient-generated signals stem from a feedback mechanism, where individuals who have experienced a service provider’s quality can offer their insights to others who lack such experience [20]. In the context of online medical platforms, these platforms allow patients to share their consultation experience with other patients and provide valuable information to those who lack first-hand experience with a hospital’s services. Such feedback acts as a critical external signal, empowering potential patients to gain knowledge about hospitals, evaluate the quality of their services, and ultimately influence their decision-making processes [28]. From a trust theory perspective, patient-generated signals contribute to the establishment of trust between potential patients and hospitals, as the aggregation of patient experiences can foster a sense of reliability and credibility. Patient-generated signals encompass two fundamental dimensions, namely, the quantity of patient reviews and the polarity of patient reviews, which reflect a hospital’s influence and reputation, respectively.

(1) Review quantity

The review quantity, which represents the total amount of patient-generated feedback, is a critical indicator of a hospital’s influence and popularity. A high review volume signals that the hospital has a large service audience, indicating its widespread acceptance and utilization by patients. When potential patients observe a substantial number of reviews, they may infer that the hospital’s services are in demand and trusted by others, which positively influences their perception of its service quality. From a trust theory perspective, a high volume of reviews enhances the perceived credibility of the hospital, as it suggests that the hospital has been widely evaluated by a wide range of patients. Empirical studies support this notion, showing that a greater volume of online reviews is positively associated with increased consultation volume in online healthcare settings. For patients considering cross-regional medical visits, a hospital with a high review volume conveys a sense of reliability and service acceptance, thereby increasing their confidence in making cross-regional medical treatment decisions. Hence, the following hypothesis is proposed:

**H2:**  *The quantity of patient reviews, as an indicator of a hospital’s influence, positively impacts patients’ willingness to choose cross-regional medical treatment.*

(2) Review polarity

The review polarity, which represents the overall positivity of patient-generated feedback, is a critical indicator of a hospital’s reputation and perceived word-of-mouth regarding its services. High review polarity, characterized by favorable ratings, positive comments, and expressions of gratitude, reflects a high level of patient satisfaction and trust in the hospital’s services [29]. When potential patients observe consistently positive feedback, they are likely to infer that the hospital provides high-quality and reliable services, thereby reinforcing its reputation and perceived value [30]. Empirical studies have demonstrated that higher polarity in patient reviews is significantly associated with increased patient trust and appointment volumes in online healthcare settings. For patients considering cross-regional medical visits, who often face greater uncertainty and additional challenges in choosing a nonlocal hospital, review polarity plays an even more decisive role. A hospital with high review polarity conveys a sense of exceptional service quality and trustworthiness, thereby increasing the patient’s willingness to seek treatment from such a hospital.

**H3:**  *The polarity of patient reviews, as an indicator of a hospital’s reputation, positively impacts patients’ willingness to choose cross-regional medical treatment.*

### 2.3. The Moderating Role of Individual Differences

The previous research has suggested that personal characteristics play a significant role in determining how users interact with information technology and information systems, including in the context of online medical platforms. This study examines two aspects of the individual differences among patients that can affect their cross-regional healthcare choices: disease severity [22,31,32,33] and medical resource availability [34].

#### 2.3.1. Disease Severity

Disease severity significantly influences patients’ reliance on external signals during medical decision-making [22]. Critically ill patients face greater risks associated with inappropriate treatment or misdiagnosis, making the decision-making process more complex and cautious. These patients often seek more reliable information to guide their choices [22]. Online platforms provide patients with access to external signals, such as hospital ratings, patient feedback, and other indicators of quality and expertise, which are crucial in reducing decision-making uncertainty, particularly for severe cases [31,33]. For critically ill patients considering cross-regional healthcare, these signals are invaluable in alleviating perceived risks, enhancing their confidence in making such decisions, and providing psychological reassurance, thereby minimizing the uncertainties associated with cross-regional healthcare choices.

(1) Disease severity and hospital rating

First, the platform-generated signals, such as hospital ratings, serve as an authoritative reflection of a hospital’s overall performance and competence. These signals are especially influential for patients with severe illnesses, who prioritize the credibility and authority of information to minimize treatment risks and guide their decisions. Critically ill patients are more likely to depend on such signals to make decisions about cross-regional healthcare. Therefore, we propose the following hypothesis:

**H4a:**  *Disease severity positively moderates the effect of platform-generated signals (e.g., hospital ratings) on patients’ decisions to seek cross-regional healthcare. Specifically, patients with more severe illnesses are more likely to be influenced by platform-generated signals and, consequently, choose cross-regional healthcare.*

(2) Disease severity and patient review quantity

Second, the quantity of patient reviews serves as a signal of the hospital’s popularity, influence, and capacity to treat similar cases. Severely ill patients often perceive a larger number of reviews as a reflection of the hospital’s extensive experience and reliability, which provides them with a sense of security through the “herd effect”. This effect may further reassure critically ill patients, enhancing their confidence in making cross-regional healthcare choices. Therefore, we propose the following hypothesis:

**H4b:**  *Disease severity positively moderates the effect of patient review quantity on cross-regional healthcare choices. Specifically, patients with more severe illnesses are likely to rely more heavily on the quantity of patient reviews when making decisions about cross-regional healthcare.*

(3) Disease severity and patient review polarity

Third, the polarity of patient reviews provides deeper insights into a hospital’s medical capabilities, service quality, and treatment effectiveness, which are particularly critical for patients facing high-risk conditions. For severely ill patients considering cross-regional visits, review polarity is crucial for assessing the potential risks and benefits associated with diagnosis and treatment. These patients are more likely to prioritize the polarity of reviews as an essential extrinsic signal when evaluating cross-regional healthcare options. In contrast, patients with less severe conditions are generally less sensitive to review polarity, as their decisions may be driven more by convenience and cost rather than perceived treatment risks. Therefore, we propose the following hypothesis:

**H4c:**  *Disease severity positively moderates the effect of patient review polarity on patients’ cross-regional healthcare choices. Specifically, patients with more severe illnesses are likely to rely more heavily on the polarity of patient reviews when making decisions about cross-regional healthcare.*

#### 2.3.2. Medical Resource Availability

In medically underserved areas, the scarcity of available medical resources significantly increases patients’ reliance on external information signals when making cross-regional healthcare choices [34]. Patients in these regions often encounter limited access to high-quality medical resources and lack personal experience or exposure to such services, which leads to greater dependence on external signals to assess hospital quality. As a result, they are more likely to rely heavily on online signals, such as hospital ratings, reviews, and rankings provided by online medical platforms. These signals are perceived as credible and authoritative sources of information, especially when contrasted with the limited availability of local medical resources. This contrast further amplifies the impact of such signals on patients’ decisions to seek cross-regional healthcare.

(1) Medical resource availability and hospital rating

In regions with limited medical resources, patients exhibit a stronger dependence on platform-generated signals, such as hospital recommendations based on comparative rankings and perceived credibility. The scarcity of high-quality local options amplifies the authority of these signals, making them a critical guide for patients lacking firsthand experience with advanced healthcare. Compared with urban patients, those from medically underserved areas are more inclined to rely on these recommendations, which significantly influences their willingness to seek cross-regional healthcare. Thus, we propose the following hypothesis:

**H5a:**  *The availability of medical resources negatively moderates the effect of platform-generated signals, such as hospital ratings, on cross-regional healthcare choices. Specifically, patients in medically underserved areas are more likely to rely on hospital ratings when making cross-regional healthcare choices.*

(2) Medical resource availability and patient review quantity

Patients in medically underserved areas often lack firsthand personal or peer experience and exposure to high-quality healthcare, making them more dependent on the experiences of others to guide their decisions. The quantity of patient feedback on online platforms serves as an indicator of hospital influence and reliability, which helps reduce the perceived risks associated with seeking care in unfamiliar regions. For these patients, particularly those from medically underserved areas, a greater volume of peer reviews significantly enhances their level of trust, encouraging them to seek care in hospitals outside their immediate area and thereby enhancing their confidence in cross-regional healthcare choices. Thus, we propose the following hypothesis:

**H5b:**  *The availability of medical resources negatively moderates the effect of patient review quantity on patients’ cross-regional healthcare choices. Specifically, the quantity of peer patient reviews has a more positive effect on the willingness of patients from medically underserved areas to seek cross-regional healthcare from distant hospitals than do urban patients.*

(3) Medical resource availability and patient review polarity

In addition to the quantity of reviews, the polarity of patient feedback becomes particularly valuable for patients in medically underserved areas. Owing to insufficient exposure to high-quality medical services and lower medical literacy, these patients rely more heavily on the polarity of feedback from other patients to evaluate the real medical capabilities of hospitals. Especially when considering cross-regional healthcare choices, positive online reputations of distant hospitals help effectively alleviate patients’ uncertainty about the potential risks associated with cross-regional healthcare, thereby enhancing their confidence in seeking treatment outside their local area. Therefore, we propose the following hypothesis:

**H5c:**  *The availability of medical resources negatively moderates the impact of patient review polarity on patients’ cross-regional healthcare choices. Specifically, the polarity of peer patient reviews has a more positive effect on the willingness of patients from medically underserved areas to seek cross-regional healthcare from distant hospitals than do urban patients.*

## 3. Materials and Methods

### 3.1. Research Context and Data Collection

One of the most popular online health platforms for Chinese patients, “Good Doctor Online”, was chosen as the data source. This platform hosts over 10,000 registered hospitals, offering a variety of online medical services to patients, including text-based consultations, telephone consultations, online inquiries, and offline navigation services. Each hospital has a dedicated profile page on the platform, providing detailed information, including hospital qualifications, departments, areas of expertise, and the number of doctors. Additionally, the profiles include records of the hospital’s online service activities, such as patient visits, consultation volumes, the total number of patients served online, as well as paid and free medical services. Based on these records, the platform generates overall ratings and recommendations for patients to consider when selecting suitable hospitals.

Patients can browse hospital profiles of interest on the basis of their medical conditions, gather information about available services, and consult doctors for basic advice. However, for certain medical conditions unsuitable for online diagnosis or for those preferring in-person care, patients can use the platform’s basic consultation features to communicate with the hospital and arrange offline visits for diagnosis, tests, or treatment. In such cases, hospitals also offer online appointment services on the platform to facilitate patients’ offline visits. After visiting the hospital, they are encouraged to leave feedback and ratings regarding their experience. This feedback system not only helps future patients make informed choices but also allows patients to continue interacting with the hospital through the platform for follow-up consultations and discussions about ongoing treatment and recovery.

We collected empirical data from the website from 17 July 2006, to 31 August 2023, based on data availability. Our initial dataset comprised 10,190 hospitals registered and providing services on the platform, with a total of 613,282 patient evaluations and feedback records following offline consultations. However, private hospitals were excluded from the analysis due to the extremely limited availability of data, which would otherwise compromise the sample size and diversity necessary for a comprehensive analysis. Additionally, patient records with missing critical information, such as diagnoses, treatment processes, or outcome assessments, were excluded to mitigate potential biases arising from incomplete data, which could otherwise distort the study results. Taking these considerations into account, we conducted data preprocessing and ultimately retained records for 1901 hospitals, along with 273,884 pieces of feedback from patients who had evaluated these hospitals.

The data available for empirical analysis in this study comprise two primary sources. The first part is derived from hospital profile information, including institutional attributes such as the hospital level, and online service metrics, including hospital ratings. The second source originates from the patient evaluation system, which includes the quantity and polarity of reviews patients have provided about the hospitals they visit, as well as individual attribute information of the patients themselves, such as the type of illness they experience, its severity, and their residential area. Considering the potential risk to privacy and confidentiality, we only used the information that was available to the general public. No user identification data, such as names and ID numbers, were used to ensure that there was no risk of sensitive information disclosure.

### 3.2. Method

A binary logistic regression analysis was conducted to identify significant factors from online medical platforms that influence patients’ decisions to seek cross-regional treatment at hospitals in other locations. Binary logistic regression is particularly suitable for this analysis because the dependent variable, cross-regional healthcare choice, is binary (1 for choosing cross-regional treatment, 0 otherwise). It allows for the estimation of the probability of this decision based on various predictor variables, including platform-generated and patient-generated signals. Moreover, this model can handle both continuous and categorical predictors, which is essential given the diverse nature of the signals examined in this study. Additionally, logistic regression allows for the interpretation of odds ratios that quantify the strength of the relationship between predictors and the outcome, providing insights into the relative importance of each independent variable in influencing patients’ decisions. In the empirical model, cross-regional healthcare choice (CRH) serves as the dependent variable, whereas three extrinsic signals reflecting the quality of medical services provided by internet hospitals are used as independent variables: one platform-generated signal, hospital rating, and two patient-generated signals, review quantity and review polarity, as represented in Equation (1):

(1) $$Logit\left(CRH\right)=\alpha_{0}+\alpha_{1}*Hosptial\_level+\alpha_{2}*Hospital\_rating+\alpha_{3}*Review\_quantity+\alpha_{4}*Review\_polarity+\varepsilon$$

The dependent variable indicates whether patients who participate in the online medical platform choose cross-regional treatment. It is measured as a binary variable, with a value of 1 indicating the choice of cross-regional treatment and a value of 0 indicating local treatment. *Hospital_rating* (*HR*) serves as an independent variable representing a platform-generated comprehensive evaluation of a hospital’s medical service capabilities and online service performance. This rating is derived from a weighted scoring system that considers multiple factors, including medical quality, service attitude, and patient satisfaction. Additionally, the platform may incorporate other metrics such as service volume, service depth, response timeliness, and response satisfaction. The weights for these components are determined based on their relative importance, which is calibrated through expert consultation and platform-specific algorithms. *Review_quantity* (*RQ*) serves as an independent variable reflecting the hospital’s online influence, which is measured by counting the number of feedback entries and reviews explicitly linked to patients’ completed offline visits, as verified through the platform’s tracking system. *Review_polarity* (*RP*) serves as an independent variable reflecting the hospital’s word-of-mouth reputation. It is measured by aggregating sentiment-weighted patient reviews. The sentiment weights are assigned as follows: positive sentiments are assigned a weight of 1, and negative sentiments are assigned a weight of −1. The aggregation process involves summing the sentiment-weighted review lengths to compute the overall polarity score for a given hospital. The final review polarity score is then normalized to account for differences in the number of reviews across hospitals. In addition, this study introduces the level of the hospital (*Hospital_level*) as a control variable that may influence patients’ cross-regional healthcare choices. Hospitals in China are classified into a 3-tier system based on their ability to provide medical care, education, and research. Accordingly, *Hospital_level* (*HL*) is measured as a dummy variable with a value of 1 for tertiary hospitals and a value of 0 for other hospitals.

Furthermore, this study incorporates two moderator variables, defined as variables that influence the strength or direction of the relationship between independent and dependent variables, namely, *Disease_severity* (*DS*) and *Medical_resources* (*MR*), to examine the moderating effects of patients’ illness severity and medical resource availability in their location on their decision to seek cross-regional treatment. The moderator variable *DS* is measured using a dummy variable, with a value of 1 representing severe illness and 0 representing mild illness. The moderator variable *MR* is measured as a dummy variable, with a value of 1 indicating abundant medical resources and a value of 0 indicating inadequate medical resources, according to the China Health Statistics Yearbook. With the inclusion of these moderator variables, the model equations are formulated as follows:

(2) $$\begin{array}{cc} Logit\left(CRH\right)=\beta_{0} & \;+\beta_{1}-Hospital\_level+\beta_{2}*Hospital\_rating+\beta_{3}\\ & \;*Review\_quantity+\beta_{4}*Review\_polarity+\beta_{5}\\ & \;*Disease\_severity+\beta_{6}*Disease\_severity\\ & \;*Hospital\_rating+\beta_{7}*Disease\_severity\\ & \;*Review\_quantity+\beta_{8}*Disease\_severity\\ & \;*Review\_polarity+\varepsilon \end{array}$$

(3) $$\begin{array}{cc} Logit\left(CRH\right)=\gamma_{0}+ & \gamma_{1}-Hospital\_level+\gamma_{2}*Hospital\_rating+\gamma_{3}\\ & \;*Review\_quantity+\gamma_{4}*Review\_polarity\\ & \;+\gamma_{5}\;Medical\_resources+\gamma_{6}*Medical\_resources\\ & \;*Hospital\_rating+\gamma_{7}*Medical\_resources\\ & \;*Review\_quantity+\gamma_{8}*Medical\_resources\\ & \;*Review\_polarity+\varepsilon \end{array}$$

## 4. Results

### 4.1. Preliminary Analysis

Table 1 presents the descriptive statistics of the independent and moderator variables. The patients were categorized into two groups: the cross-regional treatment group and the local treatment group. The cross-regional treatment group comprises 216,793 patients, accounting for 79.16% of the total population, whereas the local treatment group includes 57,091 patients, accounting for 20.84% of the total population.

On average, the hospitals visited by patients in the cross-regional treatment group have a higher level than those visited by patients in the local treatment group (88.60 vs. 11.40). Compared with the local treatment group, the cross-regional treatment group presented higher average values for hospital ratings provided by online platforms, the quantity of patient reviews, and the polarity of these reviews (4.37 vs. 4.09; 34.58 vs. 21.84; 0.33 vs. 0.31). Moreover, for the two moderator variables, patients in the cross-regional treatment group presented higher levels of disease severity but had lower average values for the availability of medical resources in their regions than patients in the local treatment group did (0.40 vs. 0.23; 0.45 vs. 0.71).

Table 2 displays the correlation matrix encompassing all the measured variables, which includes the dependent variable, the independent variables, and the moderator variables. The results show that the correlations among the variables are relatively low, suggesting the absence of significant multicollinearity concerns.

To further validate this observation, we calculated the variance inflation factor (VIF) for all explanatory variables. As shown in Table 3, the VIF values were all below 2 (Mean VIF = 1.08), confirming that there is no substantial multicollinearity in our model.

### 4.2. Hypotheses Tests

A logistic regression model was constructed to test all of the hypotheses. The basic model regressed the dependent variable CRH on the three independent variables *HR*, *RQ*, and *RP* to test the effects of the platform-generated signals and patient-generated signals indicating the quality of the online medical services provided by internet hospitals on patients’ cross-regional healthcare choices.

Table 4 presents the results of the logistic regression analysis. We found that all three independent variables are significant predictors of cross-regional healthcare choice. The detailed analyses are as follows. First, the coefficient of the independent variable *HR* is significantly positive (B = 0.406, *p* < 0.01), indicating that the online ratings for internet hospitals have a positive effect on patients’ decisions about cross-regional medical visits. Thus, Hypothesis 1 is supported. Second, the coefficient of the independent variable *RQ* is significantly positive (B = 0.089, *p* < 0.01), indicating that the volume of patient reviews has a positive effect on patients’ willingness to choose cross-regional medical treatment. Thus, Hypothesis 2 is supported. Finally, the coefficient of the independent variable *RP* is significantly positive (B = 0.634, *p* < 0.01), indicating that the polarity of patient reviews has a positive effect on patients’ willingness to choose cross-regional medical treatment. Thus, Hypothesis 3 is supported. The likelihood-ratio chi-square statistic (LRχ^2^) for the model is 52,725.12, indicating that the independent variables in the model have a strong explanatory power for the dependent variable, and the model fits the data well. The Prob > LRχ^2^ value of 0.000, which is far below 0.05, suggests that the model is statistically significant and the independent variables have a significant impact on the dependent variable. Additionally, McFadden’s R^2^ value of 0.188 suggests that the model explains approximately 18.8% of the variation in the dependent variable.

Next, the moderator variable *DS* and the interaction terms between this moderator and the three independent variables were incorporated into the baseline model to examine how the severity of a patient’s illness moderates the relationship between the external signals provided by internet hospitals and patients’ willingness to choose cross-regional medical treatment. Table 5 presents the results of the moderating effects of *DS*. First, the coefficient of the interaction term between *HR* and *DS* was significantly negative (B = −0.365, *p* < 0.01), which is the opposite of the main effect of *HR*. This finding indicates that the impact of platform-generated signals on patients’ cross-regional healthcare choices is significantly weaker for patients with high-risk illnesses than for those with low-risk illnesses. Hence, H4a was not supported. Second, the coefficient of the interaction term between *RQ* and *DS* was significantly positive (B = 0.004, *p* < 0.01), which is consistent with the main effect of *RQ*. This suggests that the volume of patient reviews has a more positive effect on patients’ willingness to choose cross-regional medical treatment in high-risk patients than in low-risk illnesses. Hence, H4b was supported. Third, the coefficient of the interaction term between *RP* and *DS* was significantly positive (B = 0.337, *p* < 0.01), which is consistent with the main effect of *RQ*. This finding indicates that the polarity of patient reviews has a more positive effect on patients’ willingness to choose cross-regional medical treatment in high-risk patients than in low-risk patients. Hence, H4c was supported. The LRχ^2^ value for this model is 53,374.42, indicating strong explanatory power. The Prob > LRχ^2^ value of 0.000 confirms that the model is statistically significant. Furthermore, McFadden’s R^2^ value of 0.190 suggests that the model explains approximately 19.0% of the variation in the dependent variable.

**Figure 1:**
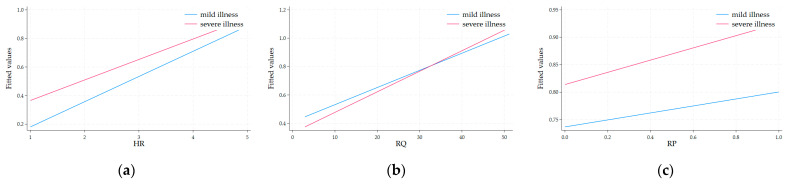
The interaction plots for the moderating effects of disease severity. (**a**) The interaction plot for the moderating effects of DS with HR; (**b**) The interaction plot for the moderating effects of DS with RQ; (**c**) The interaction plot for the moderating effects of DS with RP.

To further clarify the moderating role of disease severity, we have included Figure 1, which presents the interaction effects of DS with HR, RQ, and RP. As shown in Figure 1, the relationships between these variables vary significantly across different levels of disease severity.

Finally, the moderator variable *MR* and the interaction terms between this moderator and the three independent variables were incorporated into the baseline model to examine how the availability of medical resources in a patient’s location moderates the relationship between the external signals provided by internet hospitals and patients’ willingness to choose cross-regional medical treatment. Table 6 presents the results of the moderating effects of *MR*. First, the coefficient of the interaction term between *HR* and *MR* was significantly negative (B = −0.138, *p* < 0.01), which is the opposite of the main effect of *HR*. This finding indicates that the impact of platform-generated signals on patients’ cross-regional healthcare choices was weaker for patients from regions with abundant medical resources. In other words, patients from medically underdeveloped regions rely more heavily on platform-generated signals. Hence, H5a was supported. Second, the coefficient of the interaction term between *RQ* and *MR* was also significantly negative (B = −0.029, *p* < 0.01), which is the opposite of the main effect of *RQ*. This suggests that the volume of patient reviews has a less positive effect on the willingness of patients from regions with abundant medical resources to seek cross-regional medical treatment. Conversely, patients from medically underdeveloped regions place greater importance on the quantity of reviews provided by other patients. Hence, H5b was supported. Third, the coefficient of the interaction term between *RP* and *MR* was significantly positive (B = 0.273, *p* < 0.01), which is consistent with the main effect of *RP*. This finding indicates that the polarity of patient reviews has a stronger positive effect on the willingness of patients from regions with abundant medical resources to choose cross-regional medical treatment. As a result, H5c was not supported. The LRχ^2^ value for this model is 69,901.26, indicating strong explanatory power. The Prob > LRχ^2^ value of 0.000 confirms the model’s statistical significance. Moreover, McFadden’s R^2^ value of 0.249 suggests that the model explains approximately 24.9% of the variation in the dependent variable.

**Figure 2:**
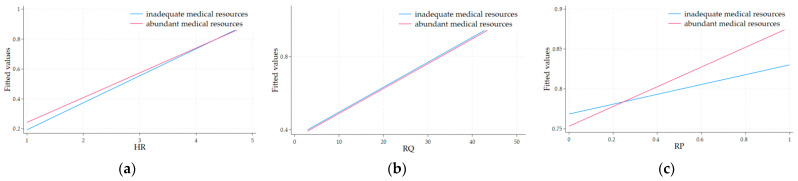
The interaction plots for the moderating effects of medical resource availability. (**a**) The interaction plot for the moderating effects of MR with HR; (**b**) The interaction plot for the moderating effects of MR with RQ; (**c**) The interaction plot for the moderating effects of MR with RP.

To further clarify the moderating role of medical resource availability, we have included Figure 2, which presents the interaction effects of MR with HR, RQ, and RP. As shown in Figure 2, the relationships between these variables vary significantly across different levels of medical resource availability.

## 5. Discussion

First, the findings of this study demonstrate that online service information from internet hospitals positively influences patients’ decisions regarding cross-regional healthcare. Online medical services offer patients a new channel through which to understand the service capabilities and quality of hospitals. This is particularly valuable for patients considering cross-regional treatment but who are anxious due to a lack of knowledge about hospitals in other areas. By effectively bridging the information gap, these platforms enable patients to gain a deeper understanding of how specific hospitals can meet their healthcare needs and address their concerns. Consequently, the availability of detailed online medical information from internet hospitals increases patients’ confidence in the target hospitals for cross-regional treatment, ultimately increasing the likelihood of their decision to seek such treatment.

Second, this study identified some critical signals from online medical services provided by internet hospitals that reflect their ability to deliver high-quality healthcare. These signals, which can be categorized as platform-generated or patient-generated, play a significant role in influencing patients’ cross-regional healthcare choices. Platform-generated signals, such as hospital ratings, serve as credibility indicators. Higher online ratings are often interpreted by patients as evidence of the hospital’s competence and expertise. This perception enhances their trust and confidence, increasing the likelihood of selecting these hospitals for treatment. As noted by Victoor et al. [12], such signals are particularly valuable in assisting patients who may lack comprehensive information or the cognitive ability to evaluate all aspects of healthcare providers systematically. Simplified cues like ratings serve as effective decision-making aids, especially for patients with limited health literacy or numeracy. Patient-generated signals include the quantity and polarity of reviews and provide further insights into the hospital’s influence and reputation. A large volume of reviews suggest that the hospital is widely recognized and influential, which helps alleviate patients’ anxiety and uncertainty about its service capabilities. This aligns with the previous studies [12], which indicate that patients rely heavily on others’ previous care experiences when making medical decisions, especially in the absence of easily accessible information. Moreover, the polarity of reviews reflects a hospital’s reputation and serves as social proof of its trustworthiness. Positive feedback from previous patients creates a sense of assurance and strengthens patients’ confidence in the hospital’s ability to deliver quality care. As Shah et al. [22] highlight, positive online word-of-mouth (WOM) not only enhances patients’ trust but also directly influences their decision-making. In conclusion, these signals have been shown to significantly shape patients’ trust and preferences when healthcare providers are selected across regions.

Third, we further explored the moderating role of disease severity and obtained some interesting findings. Specifically, patients with less severe conditions are more likely to rely on platform-generated signals, such as high online ratings of hospitals. They are likely to use this indicator as a primary reference for deciding whether to seek treatment locally or at an out-of-region hospital. In contrast, patients with more severe conditions place greater emphasis on patient-generated signals, such as the volume and polarity of patient reviews. In other words, the greater the number of reviews or the more positive the reviews from other patients are, the more likely these severely ill patients are to consider these hospitals for their cross-regional medical care. These differences suggest that disease severity significantly impacts patients’ reliance on information sources and decision-making behavior when considering cross-regional medical treatment. Possible reasons for this include the following: First, patients’ attitudes toward risk directly affect their trust in and reliance on various information sources. Severely ill patients, with heightened risk perceptions, tend to be more cautious in their decision-making process. They prefer to avoid the potential misjudgment risks associated with platform recommendations and instead rely more on authentic feedback from other patients. Such patient-generated signals are often perceived as more realistic, directly reflecting hospitals’ actual capabilities and service quality, thus making them more credible. Conversely, patients with mild conditions perceive lower health risks and are more willing to accept a certain level of uncertainty. As a result, they are less sensitive to the authenticity of signals and are more likely to trust straightforward, authoritative signals, such as platform recommendations. For these patients, platform-generated signals, which are typically based on systematic evaluations, are considered comprehensive and sufficient to facilitate quick and efficient decision-making. Second, patients with varying levels of disease severity present distinct information requirements. While platform-generated signals are authoritative, they may be perceived by severely ill patients as too generalized, lacking relevance to their specific and complex treatment needs. As a result, severely ill patients exhibit reduced reliance on platform-generated signals and seek more personalized and detailed information, which is often gathered from other patients’ reviews or alternative online and offline channels. In contrast, patients with mild conditions find platform recommendations sufficient, as these provide simple and clear guidance, enabling them to make decisions with minimal effort. Finally, from the perspective of time and effort, patients with mild conditions, owing to the lower severity of their illness, are more likely to accept platform recommendations to save time and effort without conducting extensive analyses of other signals. In contrast, severely ill patients facing greater health risks are more willing to invest considerable effort to gather and analyze a wider range of signals, such as specific feedback from other patients, to ensure they make the most informed and optimal decision possible. This aligns with the view proposed by Shah et al. [22], who argued that patients with severe conditions are more motivated to expend greater efforts and seek a broader range of information sources to ensure that they make well-informed decisions that optimize their outcomes.

Finally, we focused on the moderating role of medical resource availability and obtained some interesting findings. This study revealed that patients in regions with scarce medical resources tend to rely more heavily on platform-generated signals, such as hospital ratings, when hospitals are selected for cross-regional treatment. This reliance is partly driven by the limited spatial accessibility of offline medical resources in these regions. As Guo et al. [35] highlighted, geographic distance directly influences the availability and accessibility of medical resources, making online channels a critical tool for patients in remote or underserved areas to overcome these barriers. Additionally, these patients place greater importance on one aspect of patient-generated signals: the volume of patient reviews, as an indicator of the hospital’s influence, making them more inclined to choose such hospitals for cross-regional visits. However, a contrasting result was observed: compared with patients in areas with scarce medical resources, those in regions with more abundant medical resources tend to prioritize another aspect of patient-generated signals: the sentiment polarity of patient reviews, as an indicator of the hospital’s reputation, thus making them more likely to select these hospitals for cross-regional medical care. These differences highlight the significant impact of medical resource availability on patients’ decision-making behavior regarding cross-regional medical care, which can be explained from several perspectives. First, patients from regions with scarce medical resources prioritize the practicality of signals. They tend to rely on comprehensive information such as platform ratings and the volume of reviews, as these signals quickly reflect a hospital’s credibility and influence. This helps them reduce the cost and risk of making cross-regional healthcare choices. In contrast, patients from areas with abundant medical resources focus more on the quality of signals, especially the polarity of reviews, as they have more options and are more inclined to pursue higher-quality medical services and optimize their treatment experiences. Second, patients in areas with scarce medical resources, due to their limited experience with diverse medical options, exhibit greater trust in platform-generated signals and give more attention to hospital influence reflected by the amount of patient feedback. These signals are perceived as authoritative and reliable, enabling them to filter potential medical options efficiently. This aligns with the observation by Guo et al. [35] that patients in remote areas may rely on online channels to compensate for the lack of local familiarity with medical resources. Conversely, patients in areas with abundant medical resources, with better access to high-quality healthcare and greater experience in evaluating medical services, are more inclined to trust the authentic experiences reflected in patient reviews. They may perceive platform recommendations as overly commercialized or generalized, making them less influential in their decision-making. Finally, psychological and social factors play critical roles. Patients in areas with scarce medical resources rely more on the sense of security provided by group consensus. They tend to use platform recommendations and review volumes to gauge collective agreement (e.g., “hospitals chosen by many are likely reliable”) to reduce the psychological burden of making the wrong choice. Conversely, patients in areas with abundant medical resources, benefiting from a wider range of choices, exhibit more individualized decision-making. They emphasize personal experiences, prioritizing the online reputation of hospitals as reflected in patient reviews to identify hospitals that offer greater value and enhanced experiences.

## 6. Implications

This study contributes to the field of internet healthcare by introducing a novel perspective on how online service information provided by internet hospitals influences patients’ offline cross-regional healthcare choices. Unlike the previous studies that focused primarily on online doctor–patient interactions, including behaviors, characteristics, motivations, and benefits, this study highlights the role of platform-generated signals and patient-generated signals in reducing information asymmetry for cross-regional medical decisions. Furthermore, by leveraging signaling theory, the study develops a tailored analytical model that identifies and categorizes critical signals, offering a systematic framework to understand their differential impacts on patients’ decisions on the basis of disease severity and local medical resource availability. These insights expand our understanding of the intersection between digital information environments and offline healthcare behaviors, enriching the theoretical discourse on patient decision-making in the digital era.

This study has significant practical implications for designers and managers of online medical platforms and internet hospitals providing online medical services. By identifying the critical role of platform-generated signals (e.g., hospital ratings) and patient-generated signals (e.g., the quantity and polarity of patient reviews) in shaping cross-regional healthcare choices, online platform providers can optimize signal presentation strategies and tailor recommendations to meet the diverse needs of patients, particularly considering factors such as disease severity and local medical resource availability. For instance, platforms can improve the reliability of hospital ratings by implementing multi-source rating systems that aggregate data from patients, medical professionals, and certifications by medical authorities. Additionally, platforms could incorporate verification mechanisms to detect and filter out biased, fake, or fraudulent information, thereby enhancing the overall credibility of the rating system. Furthermore, platforms could introduce features such as personalized recommendation filters based on patient demographics (e.g., age, gender, and medical history) and disease-specific support tools (e.g., symptom checkers or virtual consultations for chronic conditions) to better serve diverse patient groups. For internet hospitals, these insights highlight the importance of enhancing online signals to build patient trust, such as improving the credibility of hospital ratings and showcasing high-quality patient reviews. This, in turn, increases the likelihood of attracting potential cross-regional patients and converting them into actual clients, thereby increasing cross-regional patient mobility. Furthermore, this study provides valuable practical guidance for policy-makers and healthcare administrators to address regional disparities in medical resources by leveraging digital platforms to improve access to reliable and trustworthy information, ultimately promoting equity and efficiency in cross-regional healthcare services. These practical contributions collectively empower stakeholders to enhance patient decision-making, optimize platform design, and foster a more equitable healthcare ecosystem in the digital era.

Furthermore, the findings provide actionable insights for policymakers and healthcare administrators to enhance the effectiveness and equity of digital healthcare platforms through specific policy interventions. First, to ensure equitable access to digital healthcare services, policies should incentivize collaborations between local governments and digital healthcare platforms, focusing on improving internet infrastructure in rural and underserved areas and providing subsidies for disadvantaged populations. Second, regulatory standards should be established to ensure the consistency, reliability, and accuracy of platform-generated information, such as hospital ratings and patient reviews. Finally, policies promoting transparency in patient-generated content can help mitigate the risks of biased or misleading information. These policy interventions enhance trust, expand access, and optimize digital healthcare platforms, enabling informed and equitable cross-regional healthcare decisions in the digital era. Such strategies are particularly relevant for addressing global health challenges, such as pandemics or chronic disease management, where cross-regional collaboration and resource sharing play a critical role. By applying these insights, low- and middle-income countries, where digital healthcare adoption is accelerating, can prioritize infrastructure development and data accuracy to bridge gaps in healthcare accessibility, promote equitable outcomes, and foster resilience in healthcare systems worldwide.

While this study provides valuable insights into the impact of online service information on cross-regional healthcare choices, certain limitations must be acknowledged. One significant limitation is the potential presence of false, manipulated, or biased reviews, which could distort perceptions and lead to misleading conclusions. Although the use of large-scale aggregated data minimizes these effects, future research should focus on developing robust methodologies, such as advanced text analysis and semantic understanding techniques, to detect and filter biased or false information. These efforts will enhance the ethical and practical validity of conclusions based on online health information. Additionally, future studies could explore the generalizability of the findings across different cultural and institutional contexts, particularly in low- and middle-income countries where digital healthcare adoption is rapidly growing.

## Figures and Tables

**Table 1:** Descriptive statistics of the independent variables and moderator variables.

Variable	Total (n = 273,884)				Cross-Regional (n = 216,793)				Local (n = 57,091)			
	Min	Max	Mean	S.D.	Min	Max	Mean	S.D.	Min	Max	Mean	S.D.
*HL*	0	1	0.98	0.12	0	1	0.99	0.08	0	1	0.96	0.20
*HR*	1	5	4.31	0.52	1	5	4.37	0.50	2.6	5	4.09	0.52
*RQ*	2.92	51.22	31.92	12.42	2.92	51.22	34.58	11.70	2.92	46.90	21.84	9.55
*RP*	0	1	0.33	0.16	0	1	0.33	0.16	0	1	0.32	0.15
*DS*	0	1	0.36	0.48	0	1	0.40	0.49	0	1	0.23	0.42
*MR*	0	1	0.50	0.50	0	1	0.45	0.50	0	1	0.71	0.46

Note: S.D. = Standard Deviation.

**Table 2:** Correlation between the dependent variable (CRH), the independent variables (HL, HR, RP, and RQ) and the moderator variables (DS and MR).

Variable	(1)	(2)	(3)	(4)	(5)	(6)	(7)
(1) *HL*	1.000						
(2) *HR*	0.048 **	1.000					
(3) *RQ*	0.186 **	0.329 **	1.000				
(4) *RP*	−0.055 **	0.082 **	0.030 **	1.000			
(5) *DS*	0.073 **	0.112 **	0.276 **	0.048 **	1.000		
(6) *MR*	0.006 **	0.032 **	−0.007 **	0.035 **	−0.008 **	1.000	
(7) *CRH*	0.120 **	0.220 **	0.417 **	0.037 **	0.143 **	−0.208 **	1.000

** Correlation is significant at the 0.01 level (two-tailed).

**Table 3:** Variance inflation factor (VIF) calculation for the independent variables (HL, HR, RP, and RQ) and the moderator variables (DS and MR).

Variable	VIF	1/VIF
*RQ*	1.23	0.810
*HR*	1.13	0.885
*DS*	1.09	0.921
*HL*	1.04	0.961
*RP*	1.01	0.987
*MR*	1.00	0.997
Mean VIF	1.08	

**Table 4:** Results of logistic regression analysis examining the impact of three independent variables (HL, HR, RP, and RQ) on the dependent variable (CRH).

Variable	Coefficient	Std. Err.	z	*p* > |z|	[95% Conf. Interval]	
*HL*	0.496	0.036	13.82	0.000	0.426	0.566
*HR*	0.406	0.011	38.25	0.000	0.385	0.427
*RQ*	0.089	0.001	170.3	0.000	0.088	0.090
*RP*	0.634	0.034	18.46	0.000	0.567	0.701
_cons	−3.578	0.056	−63.45	0.000	−3.689	−3.468
Model Evaluation						
LRχ^2^		52,725.12				
Prob > LRχ^2^		0.000				
McFadden’s R^2^		0.188				

**Table 5:** Results of logistic regression analysis examining the moderating effects of disease severity.

Variable	Coefficient	Std. Err.	z	*p* > |z|	[95% Conf. Interval]	
*HL*	0.480	0.036	13.290	0.000	0.409	0.551
*HR*	0.501	0.012	40.580	0.000	0.477	0.525
*RQ*	0.086	0.001	135.360	0.000	0.085	0.088
*RP*	0.528	0.040	13.170	0.000	0.449	0.606
*DS*	1.579	0.100	15.840	0.000	1.384	1.775
*HR × DS*	−0.365	0.024	−15.010	0.000	−0.413	−0.317
*RQ × DS*	0.004	0.001	3.110	0.002	0.001	0.006
*RP × DS*	0.337	0.078	4.310	0.000	0.184	0.491
*_cons*	−3.924	0.063	−62.540	0.000	−4.047	−3.801
Model Evaluation						
LRχ^2^		53,374.42				
Prob > LRχ^2^		0.000				
McFadden’s R^2^		0.1904				

**Table 6:** Results of logistic regression analysis examining the moderating effects of medical resource availability.

Variable	Coefficient	Std. Err.	z	*p* > |z|	[95% Conf. Interval]	
*HL*	0.567	0.038	15.050	0.000	0.493	0.641
*HR*	0.554	0.020	28.380	0.000	0.516	0.592
*RQ*	0.117	0.001	108.440	0.000	0.115	0.119
*RP*	0.635	0.061	10.330	0.000	0.514	0.755
*MR*	−0.288	0.096	−2.990	0.003	−0.477	−0.100
HR * MR	−0.138	0.024	−5.840	0.000	−0.185	−0.092
RQ * MR	−0.029	0.001	−22.940	0.000	−0.031	−0.026
RP * MR	0.273	0.075	3.640	0.000	0.126	0.420
*_cons*	−4.044	0.087	−46.360	0.000	−4.215	−3.873
Model Evaluation						
LRχ^2^		69,901.26				
Prob > LRχ^2^		0.000				
McFadden’s R^2^		0.249				

## Data Availability

The data presented in this study are available on request from the corresponding author. The data are not publicly available due to ethical and privacy restrictions.
